# Nanog/NFATc1/Osterix signaling pathway-mediated promotion of bone formation at the tendon–bone interface after ACL reconstruction with De-BMSCs transplantation

**DOI:** 10.1186/s13287-021-02643-9

**Published:** 2021-11-14

**Authors:** Kai Tie, Jinghang Cai, Jun Qin, Hao Xiao, Yangfan Shangguan, Hui Wang, Liaobin Chen

**Affiliations:** 1 Department of Orthopedic Surgery, Zhongnan Hospital of Wuhan University, Wuhan, 430071 China; 2 Department of Pharmacology, Basic Medical School of Wuhan University, Wuhan, 430071 China

**Keywords:** Dedifferentiation, BMSCs, Osteogenic differentiation, Anterior cruciate ligament, Tendon–bone healing

## Abstract

**Background:**

Bone formation plays an important role in early tendon–bone healing after anterior cruciate ligament reconstruction (ACLR). Dedifferentiated osteogenic bone marrow mesenchymal stem cells (De-BMSCs) have enhanced osteogenic potential. This study aimed to investigate the effect of De-BMSCs transplantation on the promotion of bone formation at the tendon–bone interface after ACLR and to further explore the molecular mechanism of the enhanced osteogenic potential of De-BMSCs.

**Methods:**

BMSCs from the femurs and tibias of New Zealand white rabbits were subjected to osteogenic induction and then cultured in medium without osteogenic factors; the obtained cell population was termed De-BMSCs. De-BMSCs were induced to undergo osteo-, chondro- and adipo-differentiation in vitro to examine the characteristics of primitive stem cells. An ACLR model with a semitendinosus tendon was established in rabbits, and the animals were divided into a control group, BMSCs group, and De-BMSCs group. At 12 weeks after surgery, the rabbits in each group were sacrificed to evaluate tendon–bone healing by histologic staining, micro-computed tomography (micro-CT) examination, and biomechanical testing. During osteogenic differentiation of De-BMSCs, an siRNA targeting nuclear factor of activated T-cells 1 (NFATc1) was used to verify the molecular mechanism of the enhanced osteogenic potential of De-BMSCs.

**Results:**

De-BMSCs exhibited some properties similar to BMSCs, including multiple differentiation potential and cell surface markers. Bone formation at the tendon–bone interface in the De-BMSCs group was significantly increased, and biomechanical strength was significantly improved. During the osteogenic differentiation of De-BMSCs, the expression of Nanog and NFATc1 was synergistically increased, which promoted the interaction of NFATc1 and Osterix, resulting in increased expression of osteoblast marker genes such as COL1A, OCN, and OPN.

**Conclusions:**

De-BMSCs transplantation could promote bone formation at the tendon–bone interface after ACLR and improve the biomechanical strength of the reconstruction. The Nanog/NFATc1/Osterix signaling pathway mediated the enhanced osteogenic differentiation efficiency of De-BMSCs.

**Supplementary Information:**

The online version contains supplementary material available at 10.1186/s13287-021-02643-9.

## Background

The anterior cruciate ligament (ACL) is one of the most important stable structures of the knee joint, and the meniscus and cartilage are easily injured after ACL rupture, predisposing the knee to early degenerative changes [1]. Similar to that of the articular cartilage and meniscus, the healing potential of the ACL is extremely poor [2], and reconstruction surgery is often required. Anterior cruciate ligament reconstruction (ACLR) can restore knee stability and prevent further damage to the injured knee [3, 4]. ACLR has been widely used in clinical practice, and the incidence of ACLR has significantly increased over time [5]. Although tendon grafts, including hamstring and patellar tendon grafts, have been used in ACLR for decades with satisfactory long-term clinical outcomes [6, 7], there are still some cases that require revision surgery, which is related to poor tendon–bone healing after reconstruction [8]. The success of ACLR largely depends on the biological healing between the graft and bone tunnel, and ideal ACLR using tendon grafts requires biological healing of the bone-to-tendon interface between the host bone and transplanted tendon. However, problems such as the relatively long healing time between tendon and bone and insufficient early biomechanical strength have not been fundamentally resolved. Therefore, promoting tendon–bone healing after ACL reconstruction is still an urgent problem to be solved.

To enhance the bone graft healing process, biological augmentation techniques, such as transplantation of bone marrow stem cells (BMSCs) [9], platelet-rich plasma (PRP) injection [10], and growth factor and gene transfer [11], have been applied in ACLR in recent years. It was reported that radiolabeled BMSCs were evenly distributed at the tendon–bone interface during the healing process when they were implanted into the tendon–bone interface after ACL reconstruction [12], suggesting that BMSCs might play an important role in promoting tendon-to-bone tunnel healing. In view of the role of BMSCs in tendon–bone healing and their self-renewal and multi-differentiation potential, stem cell transplantation is considered to be a promising method to promote tendon–bone healing [13], and clinical application has been attempted [14]. Bone formation plays an important role in early tendon–bone healing [15], and the biomechanical strength of the tendon–bone connection is related to the bone mass and mineralization of regenerative tissue at the tendon–bone interface [16], implying that new bone formation in the bone tunnel is essential for early healing between bone and tendon. Previous studies using BMSCs to promote tendon–bone healing showed that transplantation of BMSCs could promote direct tendon–bone healing to some extent [9, 17, 18], but new bone formation was insufficient at the tendon–bone interface.

Dedifferentiation refers to the transformation of cells from a given differentiated state to a less differentiated or stem cell-like state and leads to reacquisition of pluripotency; dedifferentiation is a cellular process associated with reentry into the cell cycle, trans/redifferentiation, or tissue regeneration [19]. Dedifferentiated cells possess certain characteristics of primitive stem cells, and the efficiency of redifferentiation is significantly improved. Dedifferentiation also occurs during the process of directed differentiation of stem cells [20]. Studies focused on the differentiation and dedifferentiation of BMSCs found that BMSCs could be reprogrammed in vitro via neuronal differentiation and dedifferentiation, with enhanced therapeutic efficacy [21]. During the process of osteogenic differentiation of BMSCs, BMSCs could be dedifferentiated after removing the induction conditions, and the resulting cells were termed dedifferentiated osteogenic BMSCs (De-BMSCs). De-BMSCs have improved osteogenic potential in vitro and exhibit great superiority in ectopic bone formation in vivo [21, 22]. These studies, which focused on De-BMSCs, provided new insights for stem cell transplantation. Because of the essential role of bone formation at the tendon–bone interface in early tendon–bone healing, De-BMSCs implantation at the tendon–bone interface could theoretically differentiate into more osteoblasts and osteocytes during the tendon–bone healing procedure, increase bone growth in the bone tunnel and promote early tendon–bone healing.

Although the osteogenic effect of De-BMSCs is significantly enhanced, its molecular mechanism is still unclear. Nanog is a newly reported transcription factor that is expressed in primordial germ cells and embryonic stem cells and plays a key role in maintaining the self-proliferation and undifferentiated state of stem cells [23]. It selectively inhibits or promotes gene expression by binding to the regulatory regions of target genes. The expression of Nanog in De-BMSCs was significantly increased [20]. Osterix is an osteoblast-specific transcription factor belonging to the SP/KLF family [24]; it is also the key transcription factor for osteogenic differentiation and can induce the expression of many mature osteoblast marker genes [25] and play a vital role in the maintenance of bone formation [26]. Nuclear factor of activated T-cells c1 (NFATc1) is an important transcription factor for osteoclast formation [27], and it regulates the differentiation of osteoclasts. It also plays an essential role in bone formation and can combine with Osterix to form a complex and cooperatively control osteoblastic bone formation [28]. Nanog could upregulate the expression of NFATc1 during the osteogenic differentiation of stem cells, thereby promoting osteogenic differentiation [29]. Therefore, we speculated that the Nanog/NFATc1/Osterix signaling pathway might mediate the significant enhancement of osteogenic differentiation efficiency in De-BMSCs.

In the current study, we confirmed the effect of De-BMSCs transplantation to promote bone formation at the tendon–bone interface after ACLR and further explored the molecular mechanism underlying the enhanced osteogenic differentiation efficiency of De-BMSCs.

## Methods

The animal experiments in this study were performed in accordance with the Guide for the Care and Use of Laboratory Animals of the US National Institutes of Health (NIH) and performed in the Center for Animal Experiments of Zhongnan Hospital of Wuhan University. The experimental design was approved by the Committee on the Ethics of Animal Experiments of the Wuhan University School of Medicine.

### Culture of rabbit BMSCs

The isolation and culture of rabbit BMSCs from the tibias and femurs of 3-week-old New England white rabbits were performed as described previously [11]. In brief, the rabbits were anesthetized and sterilized using 75% ethanol for 15 min before surgery. The femurs and tibias were harvested, and the metaphysis of the bones was dissected under sterile conditions. Bone marrow cells were collected by flushing the cavity of the femurs and tibias with Dulbecco’s modified Eagle’s medium (DMEM)/F12 medium supplemented with 10% fetal bovine serum (Gibco, Carlsbad, CA, USA), 50 mg/ml L-ascorbic acid (Sigma-Aldrich), 1% glutamine (Sigma-Aldrich), and 100 mg/ml streptomycin and penicillin (Sigma-Aldrich). The flushed liquid was centrifuged at 1200 rpm for 8 min. The supernatants were discarded, and the cell pellets were resuspended in culture medium, expanded in T-25 flasks (Cyagen Biosciences, Santa Clara, CA, USA) with DMEM/F12 medium, and incubated at 37 °C with 5% CO_2_. The medium was changed every 3 days. When the cells reached 70–80% confluence, the adherent cells were trypsinized, harvested, and expanded. Cells that had undergone three passages were used in subsequent experiments. Cells used for transplantation at the tendon–bone interface in animal experiment were acquired from different individuals and cells from different donors were used in repeated cell experiments.

### Preparation of BMSCs, Os-BMSCs, and De-BMSCs

**Fig. 1:**
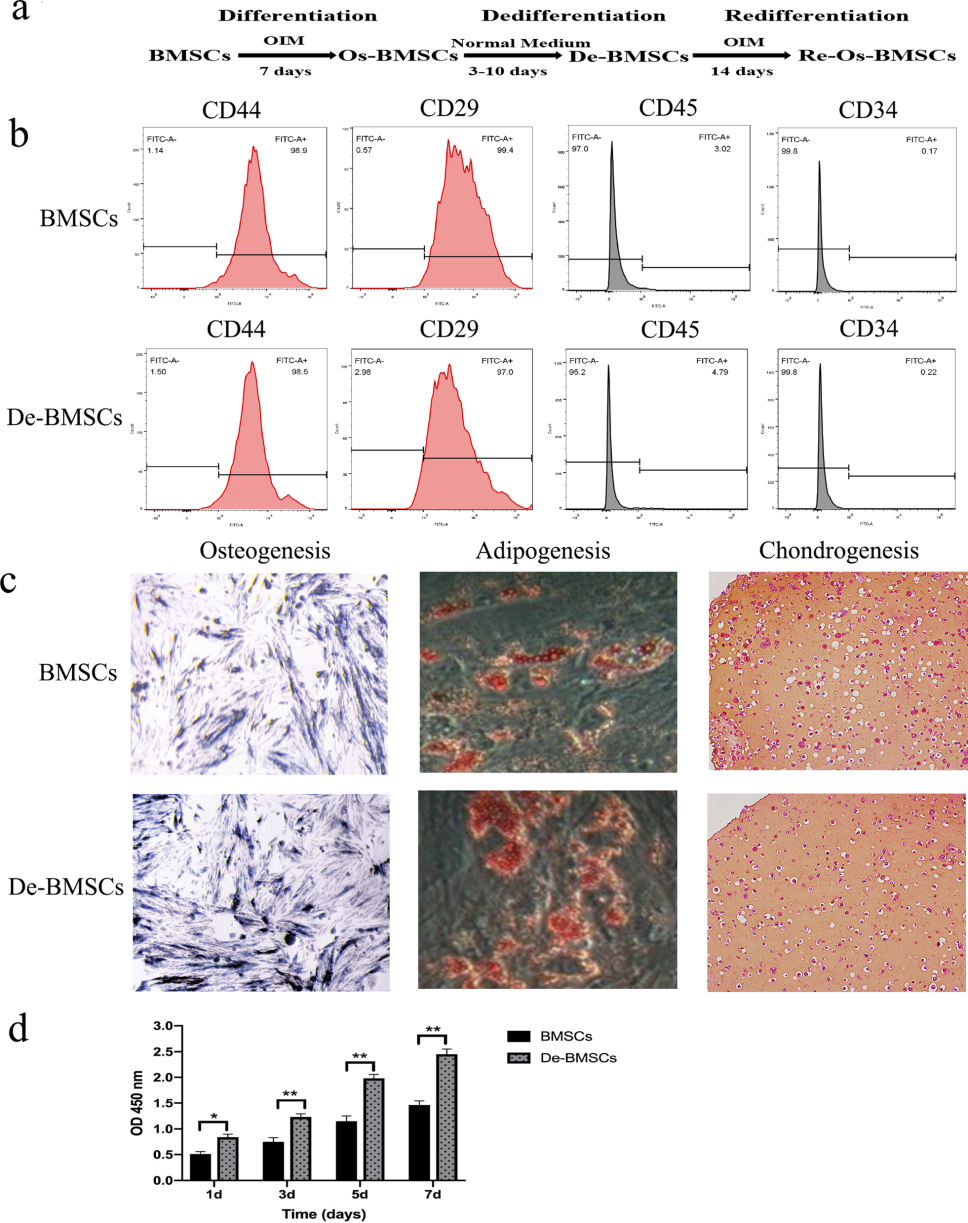
Characterization of rabbit-derived BMSCs and De-BMSCs. **a** Schematic diagram illustrating the procedure for generating De-BMSCs. BMSCs that underwent osteogenic differentiation, dedifferentiation, and redifferentiation are shown. **b** Cell surface markers of BMSCs and De-BMSCs. **c** Alkaline phosphatase (ALP), oil red O, and safranin O staining to detect osteogenesis (×40), adipogenesis (×100), and chondrogenesis (×100), respectively, of BMSCs and De-BMSCs. **d** Cell proliferation ability of BMSCs and De-BMSCs was detected by CCK-8 at 1d, 3d, 5d, and 7d (*n* = 6). *p < 0.05, **p < 0.01. *OIM* osteogenic induction media, *BMSCs* bone marrow mesenchymal stem cells, *Os-BMSCs* osteogenic bone marrow mesenchymal stem cells, *De-BMSCs* dedifferentiated osteogenic bone marrow mesenchymal stem cells, *Re-Os-BMSCs* redifferentiation osteogenic bone marrow mesenchymal stem cell

To obtain De-BMSCs, according to a previous study [22], BMSCs at p3 were transferred to osteogenic induction (DMEM)/F12 medium containing 1 nM dexamethasone, 50 μM ascorbic acid, and 20 mM β-glycerophosphate (all from Sigma-Aldrich) for 7 days. After osteogenic induction, the BMSCs were cultured in DMEM/F12 medium without inducible factors. To gain osteogenic bone marrow mesenchymal stem cells (Os-BMSCs), BMSCs were subjected to osteogenic induction medium for 7 days (Fig. 1a). In the process of dedifferentiation, dedifferentiation culture with DMEM/F12 medium was performed for 3, 7, and 10 days, and then osteogenic differentiation was performed again for 14 days. The expression of osteoblast marker genes was measured to determine the best dedifferentiation culture time for De-BMSCs, which would be transplanted at the tendon–bone interface after ACLR. The De-BMSCs were osteogenically differentiated for 24 h for investigation of the molecular mechanism.

### Cell proliferation evaluation of BMSCs and De-BMSCs in vitro

BMSCs and De-BMSCs were seeded on 96-well culture plates at a density of 2 × 10^3^ cells/well. The proliferation capacity of the two cells was measured by Cell Counting Kit-8 (CCK-8) assay (Dojinodo, Japan) at 1, 3, 5, and 7 days (*n* = 6). The medium was removed and the cells were washed with PBS. 110μL solution contained DMEM (100μL) and CCK-8 (10μL) was added to each well. Culture plates without cells were used as blanks. The absorbance was measured at a wavelength of 450 nm by microplate reader (BioTek, Winooski, VT, USA) after incubation for 1 h at 37 °C, the live cell number was correlated to optical density (OD).

### Flow cytometry (FCM) analysis of BMSCs and De-BMSCs

BMSCs and De-BMSCs were trypsinized and harvested and then incubated with fluorochrome-conjugated primary antibodies against CD29, CD34, CD44, and CD45 or the corresponding isotype control (BD Biosciences, USA) at 4 °C for 30 min. The stained cells were immediately detected using flow cytometry (BD Biosciences, USA).

### Multilineage differentiation of BMSCs and De-BMSCs

For osteogenic differentiation, BMSCs and De-BMSCs were cultured in DMEM/F12 medium containing 1 nM dexamethasone, 50 μM ascorbic acid, and 20 mM β-glycerophosphate (all from Sigma-Aldrich) for 14 days. The mineralization of BMSCs and De-BMSCs was assessed by Alizarin red S staining and alkaline phosphatase (ALP) staining. For Alizarin red S staining, BMSCs and De-BMSCs were washed with PBS, fixed with 70% ethanol for 10 min, and stained with 0.5% Alizarin red S (pH 4.1; Sigma, St. Louis, MO) for 5 min. ALP staining was performed with a BCIP/NBT alkaline phosphatase color development kit (Beyotime Institute of Biotechnology, Haimen, China) according to the manufacturer’s instructions.

For adipogenic differentiation, BMSCs and De-BMSCs were plated in a 6-well culture plate and cultured with DMEM/F12 medium containing 1 μM dexamethasone, 10 μg/mL insulin, 0.5 mM 3-isobutyl-1-methylxanthine, 0.2 mM indomethacin (all from Sigma-Aldrich, USA) and 10% FBS for 14 days [30]. Then, the cells were fixed with 70% ethanol for 10 min and stained with 0.3% fresh oil red O solution (Sigma-Aldrich) for 10 min.

For chondrogenic differentiation, BMSCs and De-BMSCs were generated following the method described in our previous work [30]. The cells were trypsinized and washed and then centrifuged at 1000 rpm for 5 min in a 15 mL polypropylene tube. The isolated cells were suspended at a concentration of 6 × 10^6^ cells/ml in 1.25% alginate (Sigma-Aldrich) in 0.15 M NaCl, and then the cell suspension was slowly dropped into a 102 mM CaCl_2_ solution. The beads were cultured in six-well plates with 5% O2 with DMEM/F12 medium containing 1% insulin, transferrin and selenium (ITS) (Sigma-Aldrich), 100 nM dexamethasone (Sigma-Aldrich), and 10 ng/ml transforming growth factor-β1 (TGF-β1) (Pepro Tech Rocky Hill, NJ, USA). On day 28, the beads were fixed in 4% paraformaldehyde for 2 h at room temperature, dehydrated in serial ethanol dilutions, and embedded in paraffin blocks. Safranin O staining was performed on paraffin sections of the beads.

### Animal study

Healthy New England Rabbits weighing 2.5–3.0 kg were purchased from the Experimental Center of the Hubei Medical Scientific Academy (Hubei, China). The rabbits were randomly distributed into the control, BMSC and De-BMSC groups (*n* = 8 for each group). BMSCs and De-BMSCs were harvested 4 weeks prior to the experiment. The mixture containing BMSCs or De-BMSCs (1 × 10^7^ cells/ml) and 1.25% alginate in 0.15 M saline was then transplanted at the tendon–bone interface of both tibial and femoral tunnels in the three groups with a microsyringe, 0.2 ml alginate gel containing 2 × 10^6^ cells were evenly injected at each time. After ACLR, in the control group, only alginate gel was injected into the tendon–bone interface; in the BMSC group, alginate mixed with BMSCs was transplanted at the tendon–bone interface; and in the De-BMSC group, alginate mixed with De-BMSCs was transplanted at the tendon–bone interface.

The ACLR model in these three groups was established following the method described by Liu et al. [31]. In brief, anesthesia was performed via ear vein injection of 30 mg/kg 3% pentobarbital. In each case, both knees were shaved, disinfected, and draped. A 4.0 cm medial parapatellar incision was made, the semitendinosus tendon was exposed and harvested at its proximal musculotendinous junction, and the tibial insertion of the tendon was preserved. The free end of the tendon was weaved with 3-0 Ethibond sutures (Johnson & Johnson) for traction. The capsule was opened from the medial side, the patellae were laterally dislocated, and the original ACL was exposed and removed. The tibial and femoral tunnels were created with a 2.5-mm drill at the footprint of the native ACL. The tunnels were irrigated with normal saline, and the weaved semitendinosus tendon was advanced through the tibial and femoral tunnels. When the tendon passed through the femoral tunnel, the free end of the tendon was sutured to the periosteum using 2-0 Ethibond (Ethicon, Somerville, NJ) in a standard fashion. BMSCs or De-BMSCs immobilized in alginate were evenly injected into the interface between the tendon and tunnel. Following implantation, the patellar retinaculum and overlying soft tissues were closed in layers. The rabbits were allowed to move their knee joints freely in their cages without restriction. An intraperitoneal injection of 4 × 10^5^ U of penicillin (Harbin Pharmaceutical Group, Shanghai, China) was administered immediately after implantation and once daily for 3 days. Knee samples were harvested from each group at 12 weeks postoperatively for further analysis. Our previous study found that t no significant differences between the data of tibial and femoral tunnel in a rabbit ACLR model [11], others also reported the same result [31]. Therefore, we only collected and analyzed the data of the tibial tunnel for the convenience of comparing.

### Histologic analysis and immunohistochemistry

The soft tissues were dissected from the knee samples, and the grafts were left intact. The knee samples were fixed in 10% formalin, decalcified in EDTA for 3 weeks, and then embedded in paraffin. Five-mm-thick sections were cut longitudinal to the bony tunnels. The slides were stained using hematoxylin and eosin. The tendon–bone interface at a depth of 5 mm from the joint surface was evaluated. The expression of osteoblast marker genes, such as COL1A, OCN, OPN, and Nanog/NFATc1/Osterix signaling pathway components, at the tendon–bone interface was analyzed by immunohistological staining. After antigen retrieval by boiling the samples in sodium citrate buffer, the sections were blocked in serum for 30 min, followed by incubation with the primary antibody in a humidified chamber at 4 °C overnight. A biotinylated secondary antibody was added for 30 min on day 2, followed by an avidin-biotinylated horseradish peroxidase complex, according to the manufacturer’s directions. Finally, peroxidase activity was revealed by immersion in DAB substrate. The following primary antibodies were used: rabbit anti-Nanog, anti-NFATc1, anti-Osterix, anti-OPN, anti-OCN, and anti-COL1A1 antibodies (all from Abcam, Cambridge, MA, USA). To characterize changes in immunostaining, the mean optical densities (MODs) were obtained from 10 areas of tendon–bone interface of tibial tunnel from 5 separate samples.

### Micro-CT analysis

Micro-CT was performed to assess the bone mass and density of newly formed mineralized tissue inside the bone tunnels. The knee samples were harvested and carefully dissected around soft tissues, with only the bone and the graft preserved. The samples were scanned by micro-CT (μCT-40, Scanco Medical, Brüttisellen, Switzerland) with the parameters set at 70 kV and 114 μA [32]. Images were obtained to assess multiple sections of the bone tunnel. Three-dimensional image reconstruction, bone tunnel area measurement, and bone volume/total volume (BV/TV) ratio analysis in the region of interest were performed. The areas of the vertical plane across the axis of the bone tunnel were measured at a depth of 5 mm from the tibial joint surface. Each area was measured three times with image analysis software (ImageJ; National Institutes of Health), and the average value was used for analysis. The region of interest was cylinder shaped, 4.0 mm in diameter, and 4 mm in length (see Additional file 1: Fig. S1).

### Biomechanics analysis

Knee samples with the femur and tibia kept at a length of 50 mm from the joint were harvested and immediately frozen at − 80 °C until testing. Before testing, the specimen was thawed overnight at room temperature. The femur–ACL graft–tibia complex was separated by resecting the attached soft tissue, and the tibial insertion of the semitendinosus tendon was also cut. Biomechanical testing was performed using a material testing machine (805, Instron Co., Norwood, MA, USA). The complex was fixed between the U-shaped clamps with 45° of knee flexion to ensure that the pulling force was parallel to the axis of the graft. The samples were preloaded with a static preload of 1 N for 5 min and then underwent the ultimate failure load test at an elongation rate of 5 mm/min. The load–deformation curve was recorded. The ultimate failure load and stiffness were determined from the load–displacement curve. The setting of biomechanical testing is shown in Additional file 2: Fig. S2.

### RNA interference

siRNAs for NFATc1 (NFATc1-rabbit-1582) were purchased from Gene Pharma (Shanghai Gene Pharma Co.). The scramble sense siRNA targeted the sequence 5’-GCCCGUAUGAGCUUCGCAUTT-3′, and the scramble anti-sense siRNA targeted the sequence 5’-AUGCGAAGCUCAUACGGGCTT-3′. In brief, De-BMSCs were plated to obtain 70–80% confluence in six-well plates and transfected with si-NFATc1 using Lipofectamine 2000 (Invitrogen, Carlsbad, CA, USA), negative control siRNA, or Lipofectamine 2000 only. After 6 h of transfection, the medium was replaced with fresh medium. The expression of NFATc1 was detected using RT-qPCR and Western blotting.

### Reverse transcription and real-time quantitative PCR

To evaluate the osteogenic differentiation potential of BMSCs and De-BMSCs, gene expression at the mRNA level was examined. Total RNA from regenerated tissues and BMSCs or De-BMSCs was extracted using TRIzol (Invitrogen) reagent following the manufacturer’s protocol. The RNA was reverse transcribed using a first-strand cDNA synthesis kit. The cDNA was amplified using a one-step polymerase chain reaction (RT-PCR) reaction. To precisely quantify gene transcripts, the mRNA level of the housekeeping gene glyceraldehyde phosphate dehydrogenase (GAPDH) was measured as the quantitative control, and each sample was normalized to the GAPDH mRNA content. The relative mRNA expression levels of Nanog, OCN, Col1A1, NFATc1, Osterix, and OPN were normalized to the level of 、GAPDH, the data was expressed as a percentage of GAPDH. The rabbit primer sequences and annealing temperatures used are shown in Table 1.

**Table 1:** Primers used for qPCR

Gene	Forward primer	Reverse primer	Annealing (°C)
GAPDH	CTCAAGATTGTCAGCAACGCA	TTGGGGGTGGGCACACGGAAG	55
Nanog	CCTGTGATTTGTGGGCCTGA	CTCTGCAGAAGTGGGTTGTTTG	55
OCN	GCCCTCACTCTTGTCGCCC	GGCTCGCTTCACCACCTCG	55
Col1A1	GCCATCAAGGTCTTCTGCG	GAACTGGAAGCCATCGGTC	55
NFATc1	CGTTCTCTCCAACACCAAGG	CTTCTCCACAAGGGGCAGTT	55
Osterix	TCAACCTCCACTGAACCCC	CCTGGTTGTAGGAGGTGGGG	55
OPN	CCTGGTTGTAGGAGGTGGGG	AGGACATAGCATTCTGCGGTG	55

*GAPDH* glyceraldehyde 3-phosphate dehydrogenase, *OCN* osteocalcin, *COL1A1* α1 chain of type I collagen, *NFATc1* nuclear factor of activated T-cells c1, *OPN* osteopontin

### Western blotting

To obtain total protein, the cells were harvested and dissolved in RIPA (Beyotime, Nanjing, China) buffer. The protein concentrations were determined with a BCA protein assay kit. Equal amounts of protein lysates (40 mg/lane) were loaded and resolved by 10% sodium-dodecyl sulfate–polyacrylamide gel electrophoresis (SDS–PAGE) (Sigma-Aldrich) and then transferred to nitrocellulose filters and probed with rabbit anti-Nanog, anti-NFATc1, anti-Osterix, anti-OPN, anti-OCN, and anti-COL1A1 antibodies at 4 °C overnight. After incubation with a horseradish peroxidase-conjugated secondary antibody (Santa Cruz Biotechnology), the blots were developed by enhanced chemiluminescence following the manufacturer’s protocol and were visualized by exposure to a Fusion FX system (Vilber Lourmat, Marne-la-Vallee, France). The protein amounts in electrophoresis gels were analyzed with Quantity One 4.6 analysis software (Bio-Rad Laboratories, Hercules, CA, USA). All solutions in this procedure contained a mixture of protease and phosphatase inhibitors.

### Immunoprecipitation

Immunoprecipitation was performed following the method described in a previous study [33]. Cell lysates were prepared in NP40 lysis buffer (50 mM Tris–HCl pH 7.4, 150 mM NaCl, 0.5% NP40, all from Sigma) or high-salt lysis buffer (20 mM HEPES pH 7.4, 10% glycerol, 0.35 M NaCl, 1 mM MgCl2, 0.5% Triton X-100, and 1 mM DTT, all from Sigma-Aldrich) with proteinase inhibitors. The supernatant was then incubated with protein G beads (GE Healthcare) and the NFATc1 antibody (Abcam, Cambridge, MA, USA) at 4 °C for 4 h. Beads conjugated with the lysates and antibodies were collected by centrifugation and washed three times with lysis buffer. The final volume of wash buffer was aspirated, and SDS loading buffer was added to the beads. The prepared proteins were resolved using 10% SDS–PAGE and then transferred to nitrocellulose membranes. Finally, the membranes were incubated with antibodies against Osterix (Abcam, Cambridge, MA, USA) for 12 h. Chemiluminescence was detected using the abovementioned ECL system.

### Statistical analysis

SPSS 17 (SPSS Science, Chicago, IL, USA) was used for data analysis. Quantitative data were expressed as the mean ± SD. One-way ANOVA followed by Dunnett’s post hoc test and Student’s t tests were used to analyze differences in the quantitative results. Statistical significance was defined as *p* < 0.05.

## Results

### Characterization of BMSCs and De-BMSCs

The general process of osteogenic differentiation and dedifferentiation is schematically illustrated (Fig. 1a). To determine whether BMSCs and De-BMSCs have stem cell characteristics, we used flow cytometry to analyze cell surface markers. The profiling showed that De-BMSCs expressed the stemness markers CD29 and CD44 but were negative for the hematopoietic markers CD34 and CD45 (Fig. 1b). The cell surface antigen profiles of De-BMSCs were similar to those of BMSCs. To determine whether De-BMSCs have the potential to differentiate into multiple lineages, osteogenic, chondrogenic, and adipogenic differentiation was induced. The results demonstrated that De-BMSCs could differentiate into osteoblasts, chondrocytes, and adipocytes, which was verified by positive staining for alkaline phosphatase (ALP), safranin O, and oil red O, respectively (Fig. 1c). The above results indicated that De-BMSCs retained stem cell properties. CCK-8 assay was used to detect the ability of cell proliferation in BMSCs and De-BMSCs, the result showed that De-BMSCs proliferated rapidly compared to BMSCs at the same time point (Fig. 1d).

### Optimal dedifferentiation culture time to promote the osteogenic differentiation potential of De-BMSCs

**Fig. 2:**
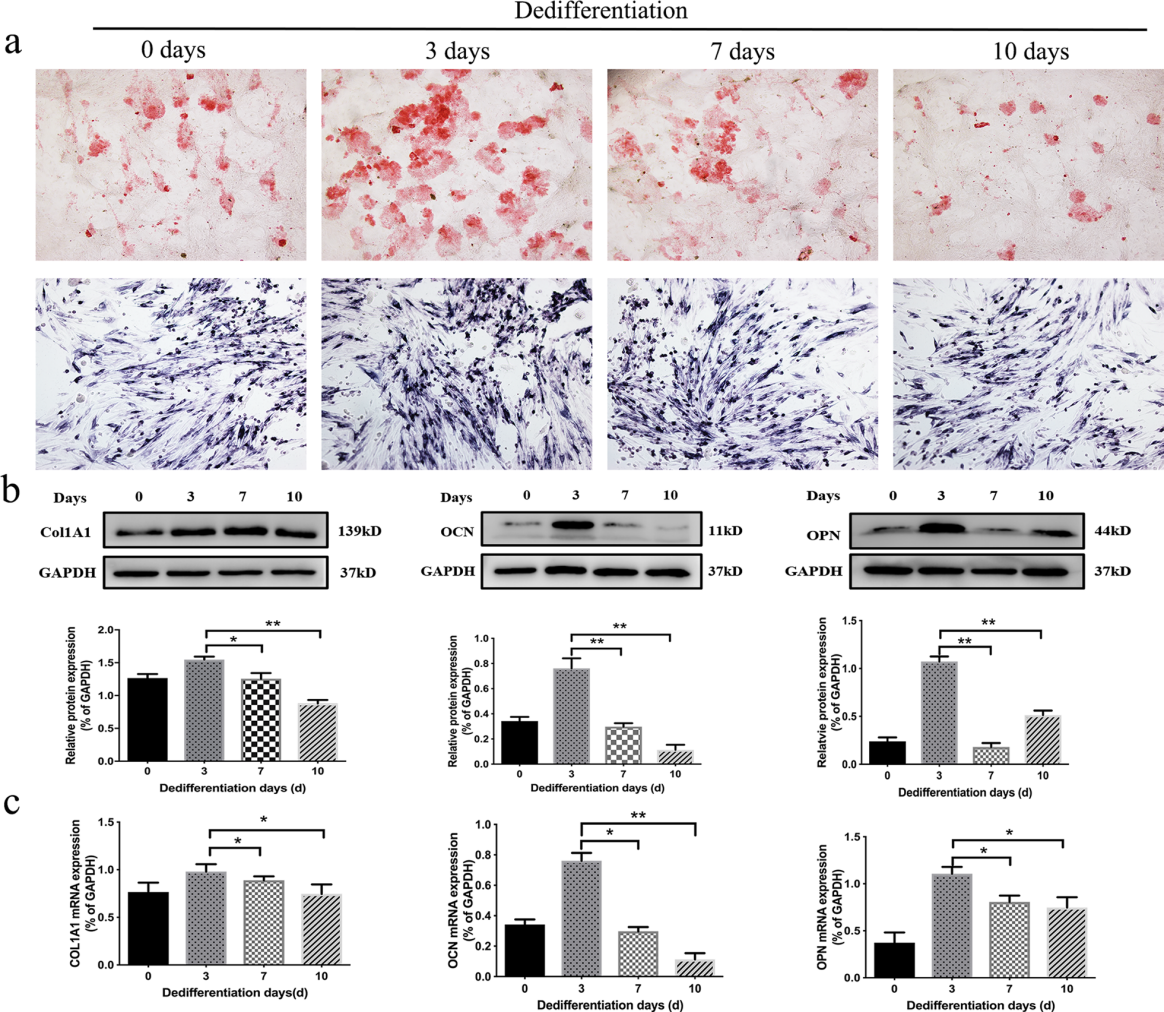
The osteogenic differentiation ability of De-BMSCs cultured for different dedifferentiation times. **a** Staining with alizarin red S (×100) and staining for alkaline phosphatase (×40) to detect the osteogenic differentiation of De-BMSCs. **b** mRNA expression of osteoblast marker genes, including COL1A1, OCN, and OPN. **c** Protein expression of osteoblast marker genes, including COL1A1, OCN, and OPN. Data represent the mean ± SD (*n* = 3). *p < 0.05, **p < 0.01. *BMSCs,* bone marrow mesenchymal stem cells; *De-BMSCs* dedifferentiated osteogenic bone marrow mesenchymal stem cells, *COL1A1* α1 chain of type I collagen, *OCN* osteocalcin, *OPN* osteopontin

It was reported that De-BMSCs had improved osteogenic potential in vitro and in vivo, and the time for dedifferentiation culture was 7 to 14 days [22, 34]. To explore the optimal dedifferentiation culture time to promote the osteogenic differentiation ability of De-BMSCs, we cultured Os-BMSCs in medium with no osteogenic factors for 3, 7, and 10 days after osteogenic induction and then transferred the De-BMSCs to osteogenic medium again for 2 weeks. The osteogenic differentiation ability of the cells was tested. Alizarin red S staining revealed that there were significantly more positive calcium nodules formed in the De-BMSCs that were dedifferentiated and cultured for 3 days (Fig. 2a). ALP staining showed similar results (Fig. 2a). We further examined the mRNA and protein expression of osteoblast marker genes after osteogenic differentiation of De-BMSCs cultured for different dedifferentiation times. The results demonstrated that the mRNA expression of COL1A1, OCN, and OPN in De-BMSCs subjected to dedifferentiation culture for 3 days was significantly increased (Fig. 2b), and the same trend was also found for protein expression (Fig. 2c). The above results indicated that the optimal dedifferentiation culture time to promote the osteogenic differentiation potential of De-BMSCs was 3 days.

### De-BMSC transplantation could promote tendon–bone healing after ACLR

**Fig. 3:**
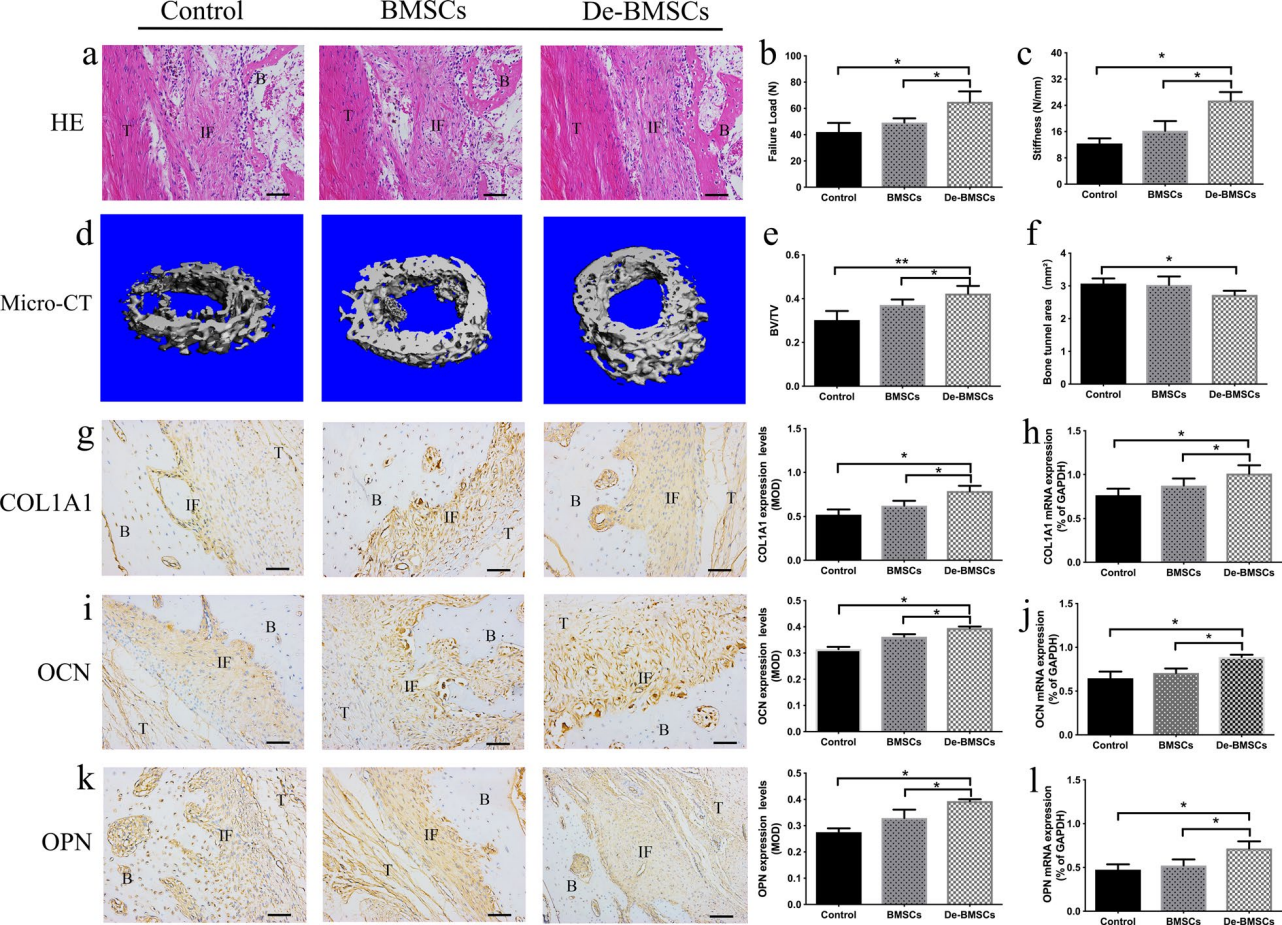
Tendon–bone healing 12 weeks after anterior cruciate ligament reconstruction (ACLR). **a** Hematoxylin and eosin staining of the tendon–bone interface. **b** Maximum failure load. **c** Stiffness. **d** Microcomputed tomography evaluation of the tendon–bone interface. **e** Bone volume/total volume (BV/TV). **f** Bone tunnel area. **g** Immunohistochemical staining for COL1A1 and quantification of the MOD at the tendon–bone interface. **h** mRNA expression of COL1A1 at the tendon–bone interface. **i** Immunohistochemical staining for OCN and quantification of the MOD at the tendon–bone interface. **j** mRNA expression of OCN at the tendon–bone interface. **k** Immunohistochemical staining for OPN and quantification of the MOD at the tendon–bone interface. **l** mRNA expression of OPN at the tendon–bone interface. Data represent the mean ± SD (*n* = 5). *p < 0.05, **p < 0.01. *Scale bar* = 50 μm. *BMSCs* bone marrow mesenchymal stem cells, *De-BMSCs* differentiation bone marrow mesenchymal stem cells, *COL1A1* α1 chain of type I collagen, *OCN* osteocalcin, *OPN* osteopontin, *MOD* mean of density, *B* bone, *IF* interface *T* tendon

Tendon–bone healing is an important factor for successful ACLR, and bone formation plays an important role in the process of tendon–bone healing [15]. In view of the enhanced osteogenic differentiation ability of De-BMSCs, we transplanted De-BMSCs that were dedifferentiated and cultured for 3 days at the tendon–bone interface after ACLR. At week 12, the rabbits in each group were sacrificed to evaluate tendon–bone healing by histologic staining, micro-CT examination, and biomechanical testing. HE staining showed that the interface zone was organized and that perpendicular collagen fibers formed crossing the junction between tendon and bone in the control group; a mature zone of cartilage was observed gradually blending into the adjacent bone and tendon, and tight fibrous tissue and a small amount of osteogenic bone tissue could be seen in the BMSC group; more newly formed surrounding bone was observed in the De-BMSC group, and there was an obvious migration connection between the newly formed bone tissue and bone tunnel (Fig. 3a). Micro-CT analysis showed more new bone formation around the tendon graft in the De-BMSC group than in the other two groups (Fig. 3d), the BV/TV value of the De-BMSC group was significantly higher than those of the control group and BMSCs group (Fig. 3e), and the bone tunnel areas of the De-BMSCs group were smaller than those of the control group (Fig. 3f). To further confirm the formation of bone tissue at the tendon–bone interface, RNA was extracted from the regenerated tissues at the tendon–bone interface of all groups. The mRNA expression of COL1A1, OCN, and OPN in the De-BMSC group was significantly increased (Fig. 3h, j, l). Immunohistochemical staining of sections with COL1A1, OCN, and OPN antibodies showed that the tendon–bone interface stained positive for these proteins. Staining for Col1A1, OCN, and OPN was stronger in the De-BMSC group than in the other two groups (Fig. 3g, i, k), which was consistent with the mRNA expression results. To clarify the effect of bone formation on tendon–bone healing after ACLR, we examined the biomechanics of the graft. Failure mode was divided into ruptures at the midsubstance or pullout from the bone tunnel. The failure pattern of the grafts was different on biomechanical test in the three groups, the majority of grafts were ruptured at the midsubstance (rupture/pullout: 4/1 grafts in Control group, 3/2 grafts in BMSCs group, and 5/0 grafts in De-BMSCs group). The maximum failure load values in the control, BMSCs, and De-BMscs groups were 42.01 ± 6.95 N, 49.24 ± 3.22 N, and 65.03 ± 7.96 N, and the stiffness values were 12.39 ± 1.56 N/mm, 16.26 ± 2.95 N/mm, 25.47 ± 2.58 N/mm. The maximum failure load and stiffness in the De-BMSC group were higher than those in the other two groups (Fig. 3b, c). These results implied that the transplantation of De-BMSCs could increase bone formation at the tendon–bone interface, thereby promoting tendon–bone healing after ACLR.

### Nanog/NFATc1/Osterix expression increased during the osteogenic differentiation of De-BMSCs

**Fig. 4:**
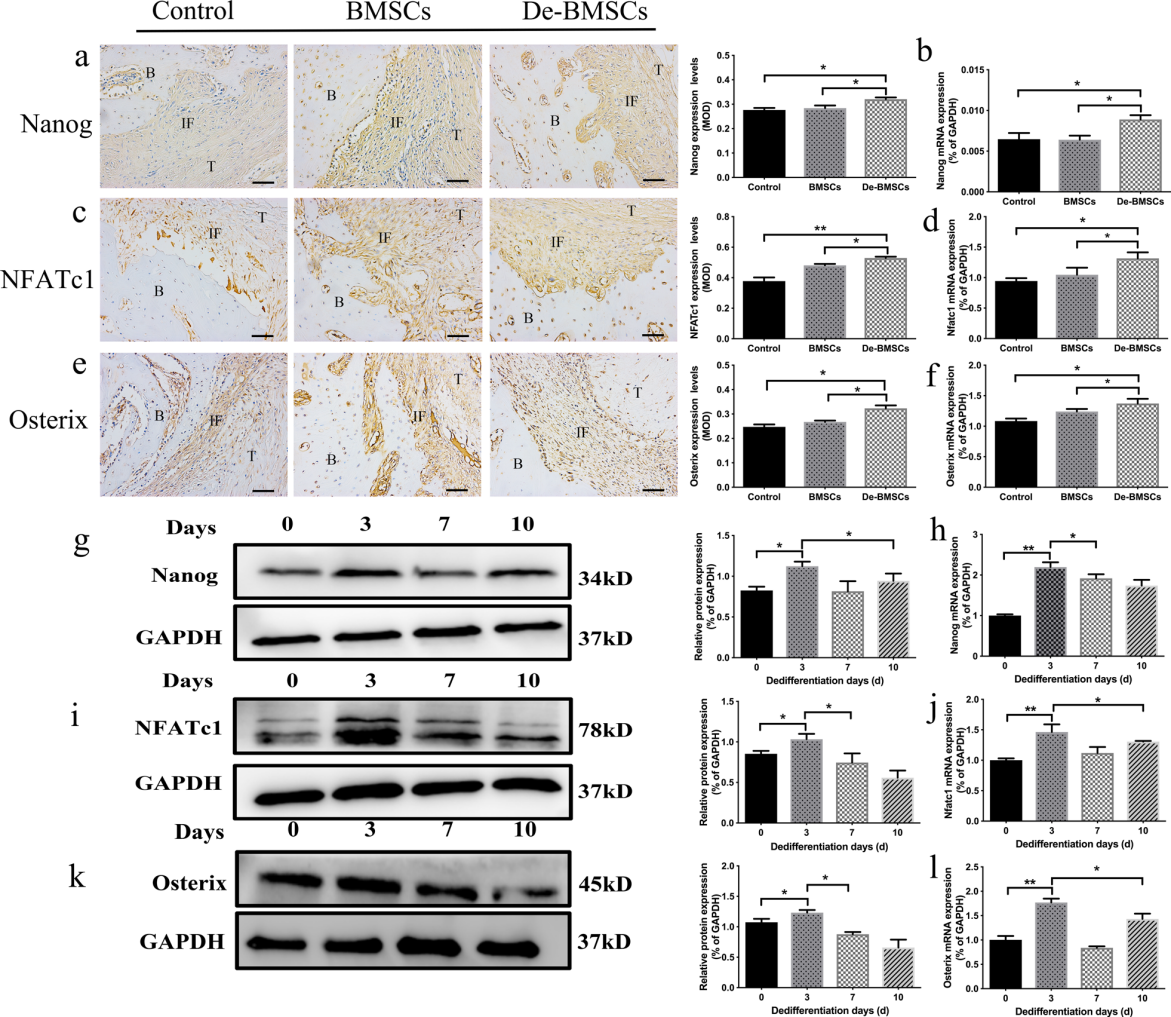
Expression of Nanog/NFATc1/Osterix signaling pathway components at the tendon–bone interface at 12 weeks after anterior cruciate ligament reconstruction (ACLR). **a** Immunohistochemical staining for Nanog and quantification of the MOD at the tendon–bone interface. **b** mRNA expression of Nanog at the tendon–bone interface. **c** Immunohistochemical staining for NFATc1 and quantification of the MOD at the tendon–bone interface. **d** mRNA expression of NFATc1 at the tendon–bone interface. **e** Immunohistochemical staining for Osterix and quantification of the MOD at the tendon–bone interface. **f** mRNA expression of Osterix at the tendon–bone interface. **g** Protein expression of Nanog. **h** mRNA expression of Nanog. **i** Protein expression of NFATc1. **j** mRNA expression of NFATc1. **k** Protein expression of Osterix. **l** mRNA expression of Osterix. Data represent the mean ± SD (*n* = 5 in vivo, *n* = 3 in vitro). *Scale bar* = 50 μm. *p < 0.05, **p < 0.01. *BMSCs* bone marrow mesenchymal stem cells, *De-BMSCs* dedifferentiated osteogenic bone marrow mesenchymal stem cells, *NFATc1* nuclear factor of activated T-cells c1, *B* bone, *IF* interface, *T* tendon

Nanog plays an essential role in maintaining the self-proliferation and undifferentiated state of stem cells [23]. Osterix is the key transcription factor for osteogenic differentiation and can induce the expression of many mature osteoblast marker genes [25]. Nuclear factor of activated T-cells c1 (NFATc1) also plays an important role in bone formation. It combines with Osterix to form a complex and regulate osteoblast differentiation [28]. Therefore, we investigated the expression of Nanog, NFATc1, and Osterix in regenerated tissues at the tendon–bone interface. The results showed that the mRNA and protein expression levels of Nanog (Fig. 4a, b), NFATc1 (Fig. 4c, d) and Osterix (Fig. 4e, f) in the De-BMSC group were significantly higher than those in the other two groups at 12 weeks after ACLR. During the osteogenic differentiation of De-BMSCs, the mRNA and protein expression levels of Nanog (Fig. 4g, h), NFATc1 (Fig. 4i, j) and Osterix (Fig. 4k, l) were also obviously increased. This result suggested that the Nanog/NFATc1/Osterix signaling pathway may play an important role in the enhanced osteogenic differentiation ability of De-BMSCs.

### The Nanog/NFATc1/Osterix signaling pathway mediated the enhanced osteogenic differentiation ability of De-BMSCs

**Fig. 5:**
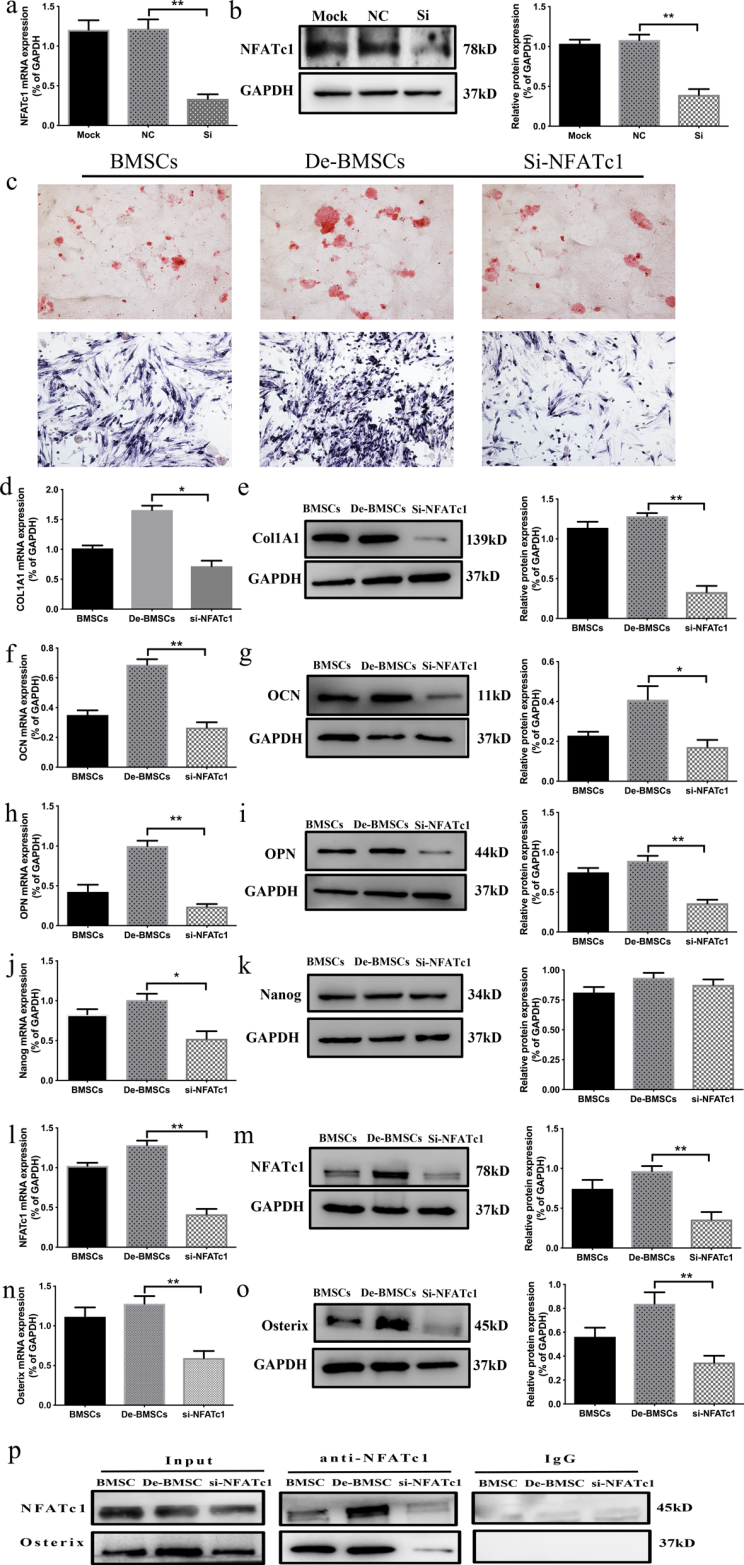
Mechanism underlying the enhanced osteogenic differentiation ability of De-BMSCs. **a** and **b** Effect of Si-NFATc1 in De-BMSCs. **c** Staining with alizarin red S and staining for alkaline phosphatase to detect the osteogenic differentiation of BMSCs and De-BMSCs. **d** mRNA expression of COL1A1. **e** Protein expression of COL1A1. **f** mRNA expression of OCN. **g** Protein expression of OCN. **h** mRNA expression of OPN. **i** Protein expression of OPN. **j** mRNA expression of Nanog. **k** Protein expression of Nanog. **l** mRNA expression of NFATc1. **m** Protein expression of NFATc1. **n** mRNA expression of Osterix. **o** Protein expression of Osterix. **p** Effects of Si-NFATc1 on the interaction of NFATc1 and Osterix. Data represent the mean ± SD (*n* = 3). *p < 0.05, **p < 0.01. *BMSCs,* bone marrow mesenchymal stem cells; *De-BMSCs* dedifferentiated osteogenic bone marrow mesenchymal stem cells, *COL1A1* α1 chain of type I collagen, *OCN* osteocalcin, *OPN* osteopontin, *NFATc1* nuclear factor of activated T-cells c1

To investigate the molecular mechanism of the enhanced osteogenic differentiation ability of De-BMSCs, we performed SiRNA-mediated stable knockdown of NFATc1 expression in cultured De-BMSCs. NFATc1 expression was significantly suppressed at both the mRNA and protein levels (Fig. 5a, b). Alizarin red S staining revealed more calcium nodules in the De-BMSC group than in the BMSC group. When si-NFATc1 treatment was performed, the number of stained calcium nodules decreased (Fig. 5c). ALP staining showed a similar result (Fig. 5c). To further verify the essential role of NFATc1 in the observed osteogenic effect, we investigated the expression of osteogenic marker genes. The results demonstrated that the mRNA and protein expression levels of COL1A1 (Fig. 5d, e), OCN (Fig. 5f, g), and OPN (Fig. 5h, i) were increased in De-BMSCs compared with BMSCs during osteogenic differentiation, and the expression of the above genes was significantly decreased when si-NFATc1 was used and lower than that in BMSCs. The above results indicated that NFATc1 plays an important role in the enhanced osteogenic differentiation ability of De-BMSCs. During the differentiation of stem cells, Nanog upregulates the expression of NFATc1, and osteoblast differentiation is regulated by the complex formed by NFATc1 and Osterix. Therefore, we examined the expression of Nanog (Fig. 5j, k), NFATc1 (Fig. 5l, m), and Osterix (Fig. 5n, o). The results showed that the mRNA and protein expression levels of Nanog, NFATc1, and Osterix in De-BMSCs were increased compared with those in BMSCs during osteogenic differentiation. After si-NFATc1 administration, the expression of NFATc1 and Osterix decreased significantly and was lower than that in BMSCs, while no significant change was observed in the expression of Nanog (Fig. 5j, k). This suggested that NFATc1 might mediate the enhanced osteogenic differentiation ability of De-BMSCs through the interaction with Osterix. To clarify this mechanism, we used Co-IP to determine whether NFATc1 interacted with Osterix. The results demonstrated that during the osteogenic differentiation of De-BMSCs, the binding of NFATc1 and Osterix was significantly increased compared with that observed during the differentiation of BMSCs, and the interaction of NFATc1 and Osterix was significantly reduced after knocking down NFATc1 (Fig. 5p). The above results indicated that increased Nanog expression upregulated the expression of NFATc1 during the osteogenic differentiation of De-BMSCs, which enhanced the interaction of NFATc1 and Osterix and subsequently increased the expression of Osterix, thereby significantly enhancing the osteogenic differentiation ability.

## Discussion

In the present study, we found that De-BMSCs could retain the characteristics of stem cells and regain the potential for multilineage differentiation. The capacity for osteogenic differentiation was improved in De-BMSCs, as indicated by increased osteoblast marker gene expression. We transplanted De-BMSCs to the tendon–bone interface after ACLR, and the results showed that more newly formed bone was observed at the tendon–bone interface than observed after transplantation of BMSCs; this change improved the biomechanical strength. It was further found that the Nanog/NFATc1/Osterix signaling pathway mediated the enhanced potential for osteogenic differentiation of De-BMSCs.

Tendon-to-bone insertion healing after ACLR is divided into direct healing and indirect healing [35]. During the process of tendon–bone healing, BMSCs infiltrate and are recruited to the interface, and a variety of cytokines are released and promote the proliferation and differentiation of stem cells to achieve tendon–bone healing [36]. Considering the role of stem cells in tendon–bone healing, many types of stem cells, such as adipose-derived mesenchymal stem cells [37, 38], BMSCs [18, 39], induced pluripotent stem cells (iPSCs) [40], umbilical cord stem cells [41], tendon-derived stem cells [42] and CD34+ ACL-derived stem cells [43], have been used to promote tendon–bone healing after ACLR. Among these stem cells, BMSCs are recognized as having the best proliferation and differentiation potential [44]. Therefore, there are many studies on the application of BMSCs to promote tendon–bone healing, and the results showed that transplantation of BMSCs could promote tendon–bone healing after ACL reconstruction, mainly indicated by increased cartilage formation at the tendon–bone interface [18, 39]. The strength of the tendon-to-bone attachment correlated with new bone formation around the tendon [45]. Although the application of BMSCs can promote healing of the tendon–bone interface, insufficient new bone formation may affect the early biomechanical strength after reconstruction.

Studies have shown that the osteogenic differentiation ability of De-BMSCs in vitro and the bone formation of De-BMSCs in vivo were increased [23, 34], but research on the application of De-BMSCs in tendon–bone healing after ACLR has not been reported. To our knowledge, this is the first report of the use of De-BMSCs to enhance bone formation at the tendon–bone interface. Micro-CT scans showed that there was more new bone generation around the graft in the De-BMSCs group than in the BMSCs group. The results of biomechanical testing showed that the maximum load and stiffness of the De-BMSC group were both higher than those of the BMSC group. These results implied that transplantation of De-BMSCs could increase new bone formation at the tendon–bone interface and enhance biomechanical strength after ACLR. Lim et al. [38] and Soon et al. [18] used BMSCs to promote tendon–bone healing after ACLR with autograft and allograft tendons, respectively. The biomechanical strength of the BMSC group in our study was similar to that reported in the abovementioned studies, and the biomechanical strength of De-BMSCs group was higher than that of BMSCs group in this study. Therefore, transplantation of De-BMSCs could increase bone formation at the tendon–bone interface after ACLR and enhance the biomechanical strength of the reconstructed ligament, which was helpful for early postoperative rehabilitation.

Although stem cells have been widely used in various regenerative medicine studies, the low cell survival rate and differentiation efficiency in vivo after transplantation has significantly reduced the effectiveness of stem cell therapy. Studies have shown that the proliferation and differentiation efficiency of De-BMSCs in vitro and in vitro is higher than that of BMSCs [23, 34], and the mechanism is mainly due to increased Nanog expression. In the present study, we also found that the expression of Nanog in De-BMSCs was increased, and its expression at the tendon–bone interface also increased after De-BMSC transplantation. Nanog is the key transcription factor controlling MSCs identity and fate conversion and plays a vital role in maintaining the self-proliferation and undifferentiated state of stem cells [24]. Nanog also plays a major role in the increased capacity for osteogenic differentiation in De-BMSCs [23], but the molecular mechanism is still unclear. Osterix is a zinc finger-containing osteoblast-specific transcription factor that can induce the expression of many mature osteoblast genes. It has been confirmed to be involved in osteoblast differentiation, maturation, and activity [46] and is also necessary for the maturation and function of osteocytes postnatally [47]. Osteogenic differentiation is a key step in osteogenesis, so Osterix also plays an important role in the process of osteogenic differentiation of stem cells. Our study showed that the expression of Osterix increased during the osteogenic differentiation of BMSCs and De-BMSCs. NFATc1 is an important transcription factor for osteoclast formation [48], and it can induce osteoclast differentiation and promote bone resorption. However, some studies have shown that the NFAT signaling pathway also plays a role in the differentiation of osteoblasts, the consequences of NFAT signaling in osteoblastic cells are controversial, and both stimulatory and inhibitory effects on osteoblastic differentiation have been reported [49]. The results of our study showed that the expression of NFATc1 was higher during the osteogenic differentiation of De-BMSCs than during that of BMSCs. The cooperation of NFATc1 and Osterix plays an important role in the formation of new bone [28]. In the present study, we found that NFATc1 interacted with Osterix during the osteogenic differentiation of BMSCs; the interaction between NFATc1 and Osterix was significantly enhanced in the osteogenic differentiation of De-BMSCs, and the expression of osteoblast marker genes was obviously increased. When siRNA was used to knock down the expression of NFATc1, the interaction between NFATc1 and Osterix was reduced, and the expression of osteoblast marker genes was decreased accordingly. During the process of osteogenic differentiation of stem cells, Nanog could promote osteogenic differentiation by enhancing the interaction between NFATc1 and Osterix, and the increased NFATc1 expression induced by Nanog might play an important role [29]. Our results demonstrated that the expression of Nanog was increased in De-BMSCs compared with BMSCs and that the expression of NFATc1 was also elevated during the osteogenic differentiation of De-BMSCs. Therefore, during the process of osteogenic differentiation of De-BMSCs, the increased Nanog expression enhanced the binding of NFATc1 and Osterix by upregulating NFATc1 and promoted the expression of Osterix at the same time; the expression of downstream osteoblast marker genes was increased accordingly, thereby enhancing the osteogenic differentiation ability of dedifferentiated BMSCs.

## Conclusions

In summary, our study found that the osteogenic differentiation ability of De-BMSCs was significantly enhanced. The mechanism was that the Nanog/NFATc1/Osterix signaling pathway increased the expression of osteoblast marker genes. Transplantation of De-BMSCs after ACLR could increase bone formation at the tendon–bone interface, thereby increasing the biomechanical strength of the reconstructed ligament. This study provides a new method for promoting tendon–bone healing after ACLR and offers new insight into the clinical application of stem cells.

## Supplementary Information

**Additional file 1. Fig. S**1: The region of interest (ROI) of tibial bone tunnel after anterior cruciate ligament reconstruction (ACLR). **a** Micro-computed tomography evaluation of the tendon–bone interface, green circles mark the bone tunnel. **b** The ROI was cylinder shaped, 4.0 mm in diameter, and 4 mm in length.

**Additional file 2. Fig. S2**: **a** The setting of biomechanical testing. **b** Load–displacement curve images of three groups.

## Data Availability

The datasets used and/or analyzed during the current study are available from the corresponding author on reasonable request.

## Abbreviations

MSCs: Mesenchymal stem cells
BMSCs: Bone marrow mesenchymal stem cells
De-BMSCs: Differentiated osteogenic bone marrow mesenchymal stem cells
Col1A1: 1 chain of type I collagen
OCN: Osteocalcin
NFATc1: Nuclear factor of activated T-cells c1
OPN: Osteopontin
DMEM: Dulbecco's minimum essential medium
RT-PCR: Polymerase chain reaction
qRT-PCR: Quantitative real-time polymerase chain reaction
GAPDH: Glyceraldehyde 3-phosphate dehydrogenase
EDTA: Ethylene diamine tetraacetic acid
SDS–PAGE: Sodium-dodecyl sulfate–polyacrylamide gel electrophoresis
HEPES: *N*-2-Hydroxyethylpiperazine-*N*-ethane-sulphonicacid
