# Supplementary figures and images for: Nanog/NFATc1/Osterix signaling pathway-mediated promotion of bone formation at the tendon–bone interface after ACL reconstruction with De-BMSCs transplantation
Source: Stem Cell Res Ther. 2021 Nov 14;12:576. doi: 10.1186/s13287-021-02643-9 (PMC8591902; doi:10.1186/s13287-021-02643-9)

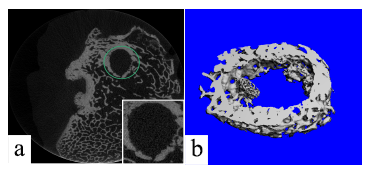

Supplement: Supplementary file 1 — Additional file 1. Fig. S1: The region of interest (ROI) of tibial bone tunnel after anterior cruciate ligament reconstruction (ACLR). a Micro-computed tomography evaluation of the tendon–bone interface, green circles mark the bone tunnel. b The ROI was cylinder shaped, 4.0 mm in diameter, and 4 mm in length. [file 13287_2021_2643_MOESM1_ESM.tif]

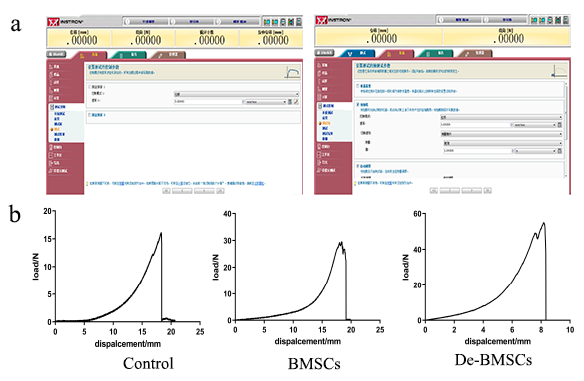

Supplement: Supplementary file 2 — Additional file 2. Fig. S2: a The setting of biomechanical testing. b Load–displacement curve images of three groups. [file 13287_2021_2643_MOESM2_ESM.tif]
